# How Professionals Share an E-Care Plan for the Elderly in Primary Care: Evaluating the Use of an E-Communication Tool by Different Combinations of Professionals

**DOI:** 10.2196/jmir.6332

**Published:** 2016-11-24

**Authors:** Catharina C de Jong, Wynand JG Ros, Mia van Leeuwen, Guus Schrijvers

**Affiliations:** ^1^ Julius Center for Health Sciences and Primary Care University Medical Center Utrecht Utrecht Netherlands; ^2^ Stichting Transmurale Zorg Den Haag eo The Hague Netherlands

**Keywords:** eHealth, primary care, elderly, email, nurses, general practitioners, medical informatics, Internet

## Abstract

**Background:**

Home-dwelling elderly patients with multimorbidity are at risk of fragmentation of care because of the many different professionals involved and a potentially unclear level of communication. Multidisciplinary communication seems to occur incidentally. Mutual feedback is needed for a professional team to provide consistent care and adequate support to the patient system. eHealth technology can improve outcomes.

**Objective:**

The aim of this study was to evaluate the use of a tool, Congredi, for electronic communication by professionals for the care of home-dwelling elderly patients.

**Methods:**

The research group was recruited through general practices and home care organizations. Congredi, a tool designed for multidisciplinary communication, was made available for professionals in primary care. It consists of a care plan and a communication channel (secure emailing). Professionals opened Congredi records for elderly patients who had 2 or more professionals involved. The records were the unit of analysis. Data were gathered from the Congredi system over a period of 42 weeks.

**Results:**

An inclusion rate of 21.4% (203/950) was achieved; nearly half of the participants were nurses. During the study, professionals were active in 448 patient records; female professionals were prevalent. In the patient records, 3 types of actions (care activities, emailing, and process activities) were registered. Most activities occurred in the multidisciplinary records (mean 12.2), which had twice the number of activities of monodisciplinary records (6.35), and solo records had a mean of 3.43 activities. Most activities were care activities (mean 9.14), emailing had a mean of 0.89 activities, and process activities had a mean of 0.29.

**Conclusions:**

An e-communication tool (Congredi) was usable for improving multidisciplinary communication among professionals. It even seemed to yield results for 40% of the professionals who used the e-care plan on their own. The content of the tool provided an active communication practice, with significant increases observed in the actions that must be shared for the effective coordination of care.

## Introduction

Worldwide the population of people older than 60 years will grow from 12% to 22% between 2015 and 2050 [1]. People over the whole world are living longer and live in their own homes as long as possible [1]. Many of the elderly will, at some point in time, need multidisciplinary professional care as a result of function loss and decreased self-care capabilities because of multimorbidity and problems in the physical, psychological, and social domains [2]. This group is at risk for the timely signaling of health risks, for aligning treatments, and for coordinating their care [2-4]. In the Netherlands in 2007, approximately 500,000 home-dwelling older persons with increased risk were identified; this is approximately a quarter of the population aged 65+ years [3]. This number is expected to increase to 1 million in 2030 in the Netherlands [5]. Among the older persons, 80% have been in recent contact with their general practitioner and half receive professional care [5]. A Dutch study shows that approximately 300,000 older persons are admitted to hospitals every year, often with nonexistent or poor multidisciplinary handover information [6]. A substantial part (20%-32%) of these hospital admissions seem to be avoidable by improving the continuity and organization of care [7]. However, because of the multidisciplinary character of care for this patient group, the care tends to be fragmented, and professionals seem to be unaware of each other’s involvement [3,8,9].

**Figure 1:**
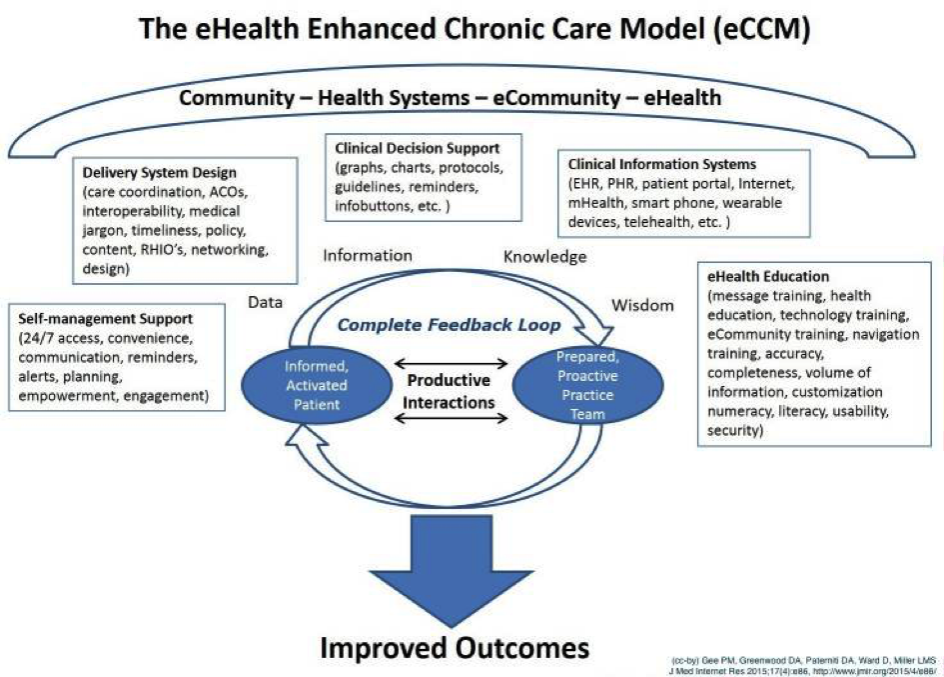
The eHealth Enhanced Chronic Care Model. ACO: accountable care organizations; RHIO: regional health information organization; EHR: electronic health record; PHR: electronic patient health record or patient portal.

The quality of primary care could improve if it were less fragmented [10]. Wagner’s chronic care model (CCM) forms a theoretical base for multidisciplinary collaboration. It focuses on a well-informed, active patient system collaborating with a prepared, proactive, and professional team to align treatment in multidisciplinary practices [2,11]. Gee et al [12] found that with the recent advancements in technology, adding eHealth options can strengthen the CCM. They developed an eHealth Enhanced Chronic Care Model (eCCM) and added a complete feedback loop between the patient system and professional team (Figure 1) [12,13]. This complete feedback loop encompasses productive interactions between the patient system and professionals about the data and information on which they can reflect from the perspectives of knowledge and wisdom by using eHealth technologies. Collaboration between the patient system and the professional team is the basis of the model, for which effective collaboration among the professionals is a precondition. Continuity and alignment of care are improved by effective communication among professionals [14,15].

Multidisciplinary collaboration in primary care is aimed at monitoring health risks and developing care plans; it is, however, unclear how such collaboration takes place [2]. The general practitioner or district nurse indicates the increasing needs of the elderly and makes an individual care plan. Usually, there are casual contacts among the involved professionals, and the contact frequency varies per case [15]. Some quantification was found in a report from 2010, which showed that for patients with diabetes and chronic obstructive pulmonary disease, multidisciplinary consultation occurs approximately once a month [10]. Communication among professionals is hampered by busy agendas, and if such contacts do take place, they are often incidental, with information being exchanged orally and not shared with others involved.

To improve the coordination of care for elderly and chronically ill patients, eHealth tools show potential, such as the sharing of care plans and online health communities [12,16-18]. Health care providers in The Hague realized this and started experimenting with a communication tool developed by a general practitioner, Congredi (Convenient Fastguide BV) in 2012 [19]. They surmised that the coordination of care would benefit if multidisciplinary communication increased. In 2013, a feasibility study of Congredi was performed on a sample in 2 neighborhoods. This showed that Congredi lived up to the original functional specifications and that professionals were motivated to continue exploring the use of Congredi. Also, a larger number of professionals than expected took part because of active early adopters who inspired their colleagues (41 instead of the expected 15). They were motivated to continue in cocreation as they had important requirements to be included in the new version of the tool and the supplier was perceived as cooperative [20]. An important requirement for these professionals was a link to their own administration system; adjustments in this area were made in the next release of Congredi, which was used for this study. The question was then raised whether an electronic communication tool for professionals could improve multidisciplinary communication and whether this would affect the integration of care. A precondition is that such a tool is actually used by professionals.

The aim of this study was to evaluate the use of a tool for electronic communication and coordination (Congredi) by professionals in the care of home-dwelling elderly patients.

## Methods

### Design

In this descriptive study, data were gathered from the Congredi system over a period of 10 months (42 weeks) and analyzed.

The following research questions were addressed:

1. How many and which professionals are linked to Congredi records?

2. How many and which actions are performed by the professionals in Congredi records?

3. Is there a relationship between the combination of professionals in the care plan and performed actions?

### Intervention

Congredi is a communication tool that was designed for multidisciplinary communication among professionals in primary care [19]. It is an easily accessible Web-based application and is compatible with existing health information technology but can also function as a stand-alone solution. It can be used on mobile phones, tablets, and computers. Congredi consists of a care plan that is usable at any moment in time. Within the care plan tasks can be delegated and feedback is received immediately. In addition, there is a communication channel (secure emailing) so the professionals can communicate asynchronously and at their own convenience.

**Figure 2:**
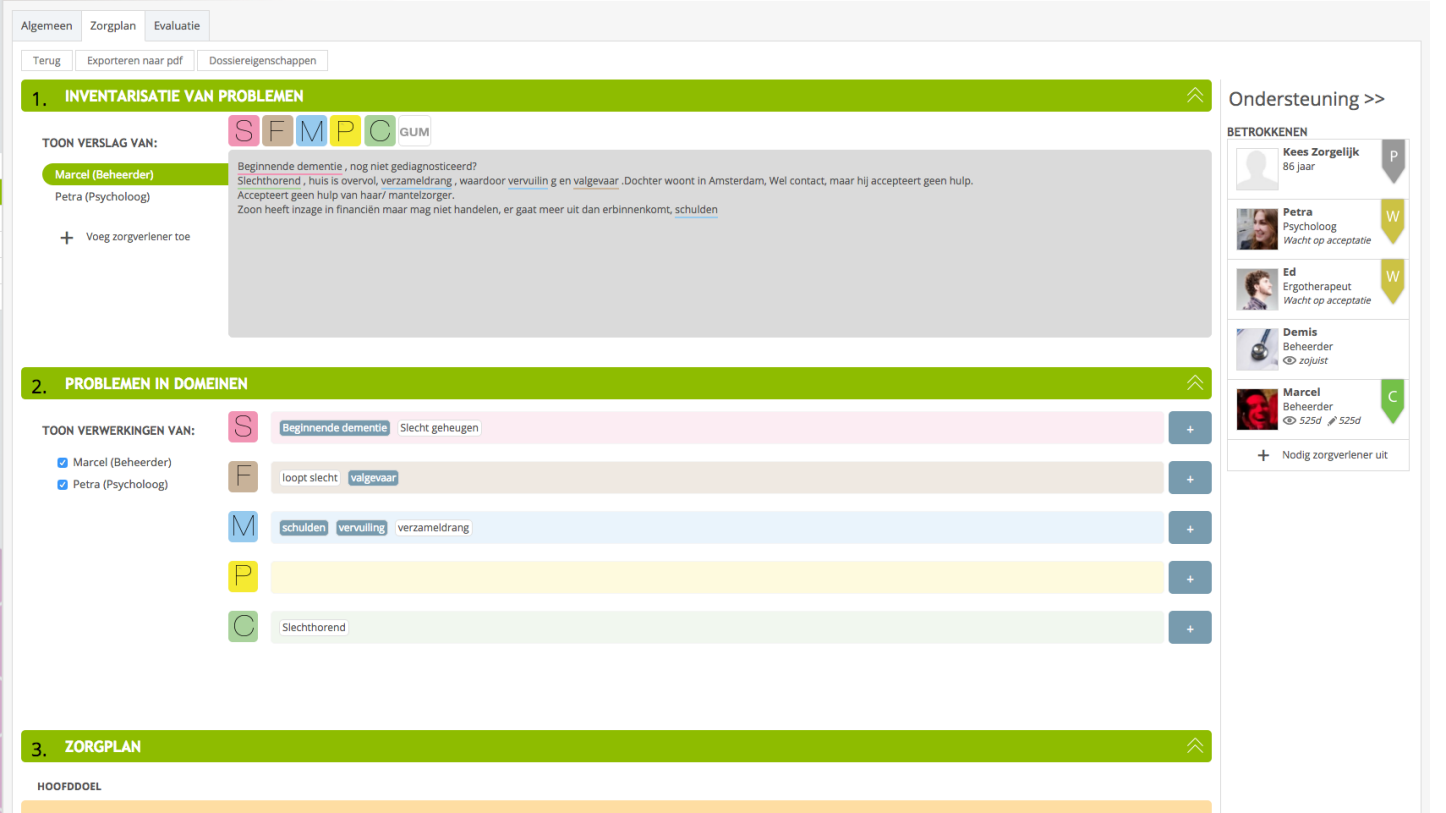
Congredi problem inventory: problems listed in text and in the social, functional, mental, physical, and communication (SFMPC) domains.

**Figure 3:**
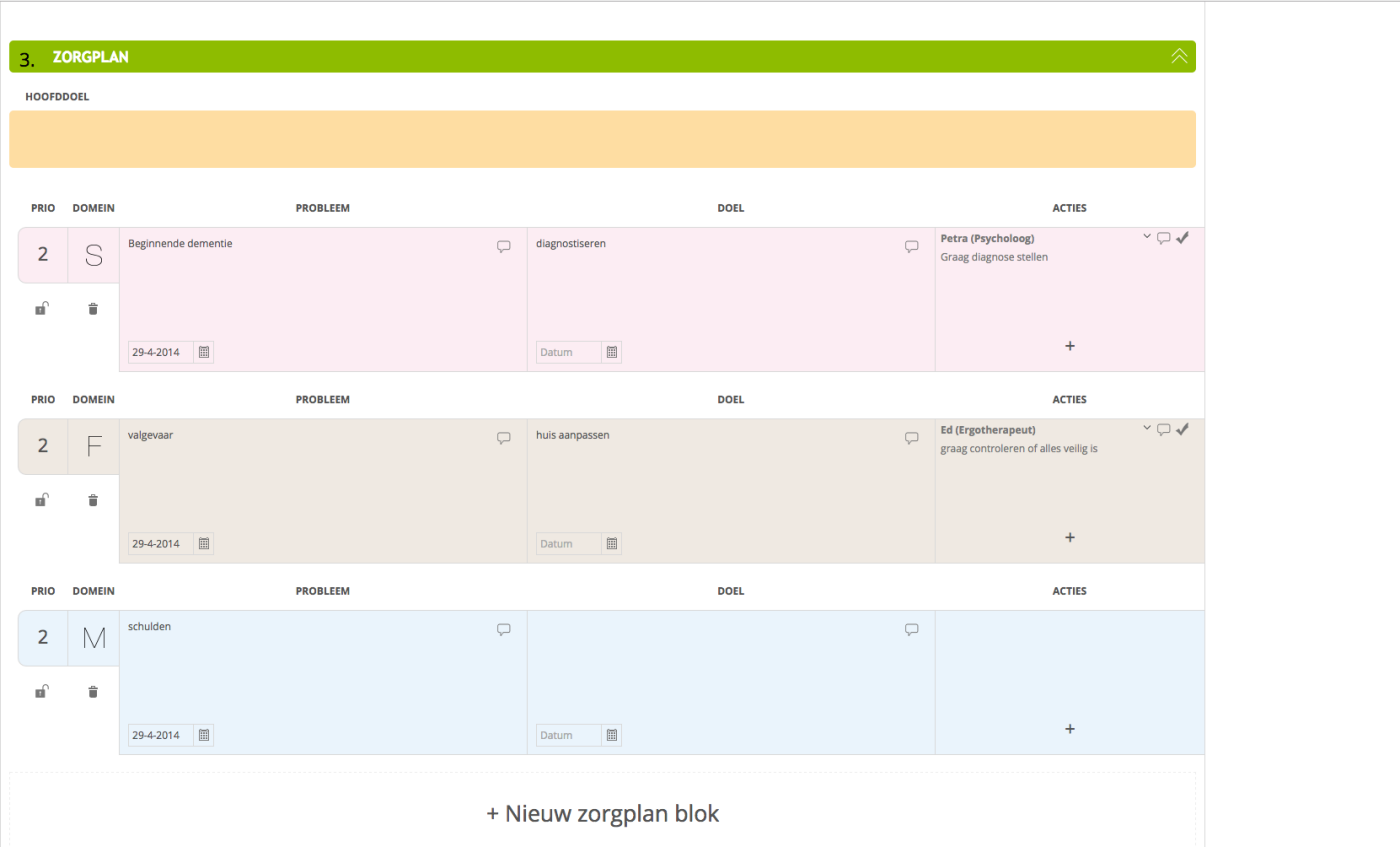
Congredi care plan: problems, aims and actions shown in social, functional, mental, physical, and communication (SFMPC) action blocks.

**Figure 4:**
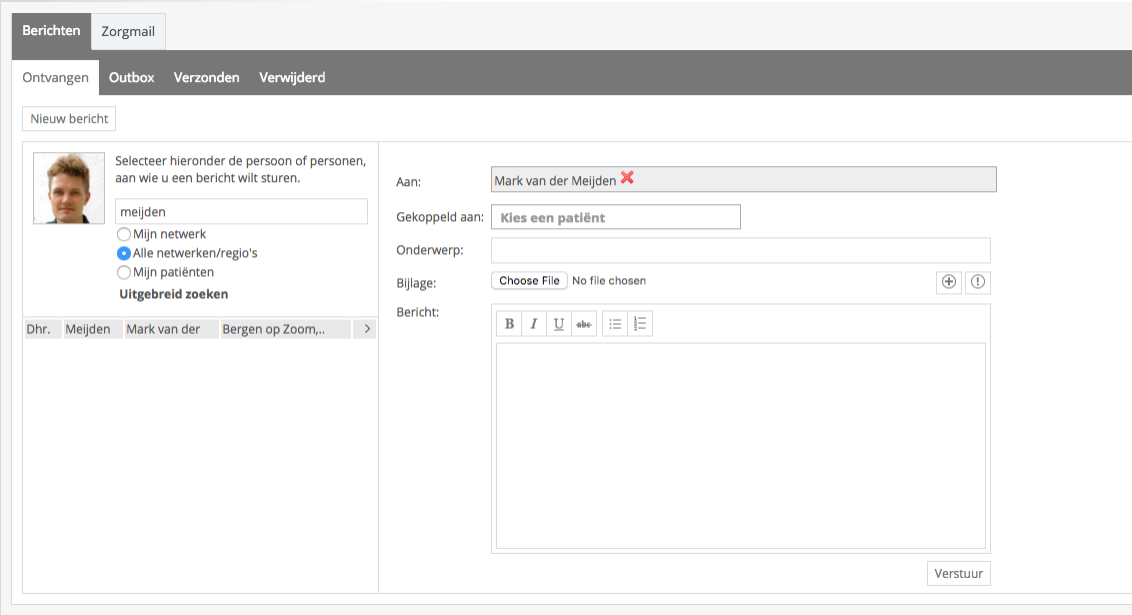
Congredi email module: secure email for professionals about their patients.

To start Congredi, a professional opens a record for a patient and starts making a care plan, which is based on the patient-centered SFMPC (social, functional, mental, physical, and communication) domain model [8]. The professionals involved with this patient can be invited to link and can thus view the record, including the shared care plan. The activities that the professionals perform within the patient records are grouped into 3 categories. First, there are care activities, which consist of the following: (1) assessment of the current problems, structured by applying colors to current problems and automatically organizing according to SFMPC domain (Figure 2); (2) care actions, actions needed to address the problems of the patient (Figure 3); (3) observations of the care process and evaluation; and (4) care action adaption is performed after evaluating the care actions. Second, there is communication by secure emailing for sending and receiving emails to colleagues within Congredi (Figure 4). The content of the emails is only visible to those directly involved. Third, some process activities are also registered, namely, (1) becoming a coordinator, as it is possible to change the person who coordinates the record; a general practitioner occasionally starts the record and later “hands over” to the nurse; and (2) inviting involved professionals to link, which can occur at different moments in time during the care process. Congredi operates alongside the monodisciplinary electronic health records of the diverse professionals; it makes patient-related communication about current multidisciplinary problems possible.

Because of multidisciplinary communication, all professionals can update the care plan as the care develops. Thus, professionals are informed about the actions of their colleagues. One professional coordinates the record and is responsible for linking other professionals.

### Research Group and Recruitment Procedure

The intervention Congredi was introduced to facilitate multidisciplinary communication about mutual patients at any time and place that was convenient to each professional. For this study, all general practitioners (n=300) and home care organizations with district nurses (n=650 nurses) in The Hague region were approached to participate; digital media were used, and the directors of home care organizations were approached personally. Professionals entered the study by applying for access to Congredi via their managers; they were then able to log-in to Congredi and received a standard half-day training.

They were then able to open a Congredi record for each patient in their care. The criteria for the patients were that they were home-dwelling elderly patients with 2 or more professional health caregivers. Patients had to give permission to open a Congredi record and share their care plan with other professionals.

Various types of professionals could participate in Congredi. In this study, we distinguished 3 groups of professionals: nurses (N), general practitioners (G), and other professionals (O). Others could be physiotherapists, psychiatrists, geriatricians, social workers, and elderly consultants.

### Variables and Measures

Data were retrieved from the Congredi system at the end of the observation period, after 10 months (42 weeks), to answer the following research questions:

1. How many and which professionals are linked to patient records?

2. How many and which actions are performed in care plans?

3. Which relationship exists between combinations of professionals and performed actions in patient records?

The following variables were measured: (1) characteristics of health care professional using Congredi, that is, demographic data (age, sex), discipline (general practitioner, nurse, other professional), and whether coordinator of patient record (yes or no); (2) characteristics of patients in Congredi, that is, demographic data (age, sex); (3) multidisciplinary combinations of health professionals in Congredi, namely, coordination of patient record, combinations of health care professionals linked in a patient record, and number of health care professionals linked to each patient record; and (4) activities performed by health care professionals in Congredi, that is, frequency of activities (care, email, and process activities) and period in which activities took place per record (number of weeks).

### Statistical Analysis

The results were analyzed using IBM SPSS 20 (IBM Corporation). The unit of analysis is the Congredi record of a patient (patient record). The demographic statistics of the population are described in frequencies and percentages. Analyses of variance, including Bonferroni post hoc tests, were performed to examine mean differences between subgroups.

## Results

### Characteristics of Professionals and Patients

Of the 300 general practitioners and 650 nurses who were approached to participate, 21.4% (203/950) actually took part. Among the professionals, 75.9% (154/203) were female. The age group between 30-50 years was 49.3% (74/203).

Nearly half of the participating professionals were nurses (47.3%, 96/203); these included different types of nurses active in primary care, such as district nurses, case managers for dementia, and nurse specialists. General practitioners (19.2%, 39/203) and other professionals (33.5%, 68/203), including elderly consultants, physiotherapists, gerontologists, and social workers, were also active in Congredi.

In total, professionals opened 532 patient records. Each patient record had a coordinator; the coordinator was a nurse in 80% (423/532) of the patient records, a general practitioner in 16% (75/532), and other professionals in 4% (33/532).

In 84 records, no further action was taken. In the remaining 448 patient records, actions were taken. Within these records, more than half of the patients were female (63%, 282/448). The largest age group was 80-90 years (45.1%, 202/448), and 13.9% (62/448) of the patients were older than 90 years.

The number of weeks the professionals were active in Congredi varied: 37.9% (77/203) were active between 1 and 26 weeks and the rest were active between 27 and 42 weeks. A total of 32.5% (66/203) stopped within a week.

### Combinations of Professionals and Level of Action in Patient Records

Several combinations of professionals (Table 1) were found to be active in patient records. “Active” was defined as taking 1 or more actions within a patient record. On the basis of the participation of professionals, 3 types of patient records could be distinguished. The first type is referred to as “solo” in which 1 professional was linked; 41.1% (184/448) of the records were solo records. The second type of patient record was “mono” in which at least 2 professionals of the same discipline were linked; 14% (63/448) were monodisciplinary records. The third type was named “multi” with professionals from different disciplines; 44.9% (201/448) were multidisciplinary records.

In the multidisciplinary records, a nurse’s participation was the most, that is, in 96.5% (194/201) of the records. This was followed by participation of general practitioners (81.6%, 164/201) and other professionals (36%, 73/201). Both the solo and monodisciplinary records consisted primarily of nurses (80.9%, 149/184 and 88.9%, 56/63, respectively). In the multidisciplinary records, the most frequent combination of professionals was general practitioner-nurse (GN 63.7%, 128/201), followed by the combination nurse-other professional (NO 18.4%, 37/201) and the combination general practitioner-nurse-other professional (GNO 14.4%, 29/201).

### Activities Undertaken by Multidisciplinary Combinations in Patient Records

In the Congredi records, 3 types of professional actions (care activities, emailing, and process activities) were registered. Most activities occurred in the multidisciplinary patient records, with a mean number of 12.2 activities per record (Table 1). When professionals worked in monodisciplinary patient records, the mean number of activities was 6.35, and in solo patient records the mean number was 3.43.

Table 2 presents the relation between the activities performed in patient records (care, email, and process activities) and the multidisciplinary combinations of professionals who performed them. Multidisciplinarity was related to the level of activity.

Problem assessment, which takes place at the beginning of a care process, was found in 84.1% (169/201) of the patient records; in most cases it was performed once (53%, 107/201), with a mean number of 1.26. Care actions, which are planned on the basis of problem assessment, were registered in 72.6% (146/201) of the patient records; in nearly 50% (95/201), care actions occurred more than once (mean 1.72). Observations, which occur between evaluative notes during the care process, were registered in 97% (195/201) of the patient records, mostly in records in which nurses were active (mean 4.09). Care action adaption, which takes place in relation to the goal of the care process, was found in 70% (141/201) of the patient records (mean 2.07). Emailing was used in 31.4% (63/201) of the patient records (mean 0.89). Handing over coordination to a colleague was registered in 28.4% (57/201) of the patient records. Inviting involved colleagues to link occurred a mean 1.88 times, ranging from 1 to 8.

**Table 1:** Combinations of health care professionals collaborating in patient records (N=448).

Category	Combination^a^	Combination, n (%)	Actions, mean	Actions, SD	Actions, minimum	Actions, maximum
Solo	Total	184 (41.1)	3.43	2.30	1	13
	G	23 (12.5)	1.91	1.20	1	7
	N	149 (80.9)	3.68	2.39	1	13
	O	12 (6.5)	2.40	1.14	1	5
Monodisciplinary	Total	63 (14.0)	6.35	3.78	1	22
	G	5 (7.9)	2.40	1.14	1	4
	N	56 (88.9)	6.64	3.79	2	22
	O	2 (3.2)	8.00	1.41	7	9
Multidisciplinary	Total	201 (44.9)	12.20	11.25	2	95
	GNO	29 (14.4)	21.21	14.07	7	54
	GN	128 (63.7)	9.66	7.38	2	45
	NO	37 (18.4)	15.03	15.89	4	95
	GO	7 (3.5)	7.77	2.99	3	11
Combination including	Total	201				
	G	164 (81.6)				
	N	194 (96.5)				
	O	73 (36.3)				

^a^G: general practitioner; N: nurse; O: others.

**Table 2:** Activities in multidisciplinary patient records by different combinations of professionals.

Activities		Frequency or mean	Total (N=201), n (%)	GNO^a^ 29 (14.4), n (%)	GN^b^ 128 (63.7), n (%)	GO^c^ 7 (3.5), n (%)	NO^d^ 37 (18.4), n (%)	*P* value
**Care activities**								
	Total care activities	Mean	9.14	15.79 (GN, GO)^e^	6.87 (GNO, NO)	4.86 (GNO)	12.62 (GN)	<.001
	Problem assessment	Mean	1.26	1.90 (GN, GO)	1.06 (GNO, NO)	0.71 (GNO)	1.54 (GN)	<.001
		0	32 (15.9)	1 (3.4)	27 (21.1)	2 (28.6)	2 (5.4)	
		1	107 (53.2)	13 (44.8)	69 (53.9)	5 (71.4)	20 (54.1)	
		2 and 3	59 (29.4)	12 (41.4)	32 (25.0)	0 (0.0)	15 (40.5)	
		>4	3 (1.5)	3 (10.3)	0 (0.0)	0 (0.0)	0 (0.0)	
	Care action	Mean	1.72	3.59 (GN, GO, NO)	1.31(GNO)	1.14 (GNO)	1.78 (GNO)	<.001
		0	55 (27.4)	1 (3.4)	45 (35.2)	2 (28.6)	7 (18.9)	
		1	51 (25.4)	4 (13.8)	34 (26.6)	2 (28.6)	11 (29.7)	
		2 and 3	70 (34.8)	12 (41.4)	39 (30.5)	3 (42.9)	16 (43.2)	
		>4	25 (12.4)	12 (41.4)	10 (7.8)	0 (0.0)	3 (8.1)	
	Observations	Mean	4.09	7.24 (GN, GO)	3.21 (GNO, NO)	2.00 (GNO)	5.08 (GN)	.006
		0	6 (3.0)	0 (0.0)	5 (3.9)	1 (14.3)	0 (0.0)	
		1-3	124 (61.7)	11 (37.9)	92 (71.9)	6 (85.7)	15 (40.5)	
		4-6	35 (17.4)	6 (20.7)	18 (14.1)	0 (0.0)	11 (29.7)	
		>6	36 (17.9)	12 (41.4)	13 (10.2)	0 (0.0)	11 (29.7)	
	Care action adaption	Mean	2.07	3.07	1.28	1.00	4.22	.07
		0	60 (29.9)	3 (10.3)	47 (36.7)	3 (42.9)	7 (18.9)	
		1	49 (24.4)	5 (17.2)	35 (27.3)	1 (14.3)	8 (21.6)	
		2 and 3	69 (34.3)	11 (37.9)	38 (29.7)	3 (42.9)	17 (45.9)	
		>4	23 (11.4)	10 (34.5)	8 (6.3)	0 (0.0)	5 (13.5)	
**Emailing**								
	Emails sent	Mean	0.89	1.83	0.70	0.43	0.89	.13
		0	138 (68.7)	16 (55.2)	94 (73.4)	5 (71.4)	23 (62.2)	
		1	33 (16.4)	4 (13.8)	22 (17.2)	1 (14.3)	6 (16.2)	
		2 and 3	18 (9.0)	4 (13.8)	8 (6.3)	1 (14.3)	5 (13.5)	
		>4	12 (6.0)	5 (17.2)	4 (3.1)	0 (0.0)	3 (8.1)	
**Process activities**								
	Becoming coordinator	Mean	0.29	0.31	0.32	0.14	0.19	.39
		0	144 (71.6)	21 (72.4)	87 (68.0)	6 (85.7)	30 (81.1)	
		1	56 (27.9)	7 (24.1)	41 (32.0)	1 (14.3)	7 (18.9)	
		2 and 3	1 (0.5)	1 (3.4)	0 (0.0)	0 (0.0)	0 (0.0)	
	Invite involved professionals to link	Mean	1.88	3.28 (GN, GO, NO)	1.77 (GNO)	1.00 (GNO)	1.32 (GNO)	<.001
		0	15 (7.5)	0 (0.0)	9 (7.0)	1 (14.3)	5 (13.5)	
		1	74 (36.8)	5 (17.2)	43 (33.6)	5 (71.4)	21 (56.8)	
		2 and 3	95 (47.3)	13 (44.8)	71 (55.5)	1 (14.3)	10 (27.0)	
		>4	17 (8.5)	11 (37.9)	5 (3.9)	0 (0.0)	1 (2.7)	

^a^GNO: general practitioner, nurse, and other professional.

^b^GN: general practitioner and nurse.

^c^GO: general practitioner and other professional.

^d^NO: nurse and other professional.

^e^The codes in parentheses (eg, GN, GO) indicate the groups with a significant mean score.

## Discussion

### Principal Findings

In this study, the use of a tool for electronic communication and coordination (Congredi) by professionals in the care of home-dwelling elderly patients was evaluated. The evaluation underscores the usability of Congredi for professionals in primary care because a large group of professionals (n=203) were active in 532 patient records. Three research questions were examined.

To answer the first question, “How many and which professionals are linked to Congredi records?” a total of 203 professionals were identified, at an inclusion rate of 21.4% (203/950). Nurses represented the largest discipline at approximately half of the sample, besides general practitioners, and various other disciplines.

The second question was “How many and which actions are performed in Congredi records?” To answer this question, the patient records were divided into 3 categories. Patient records in which professionals worked on their own were defined as solo records (184/448, 41.1%). When several colleagues of the same discipline were linked, this was considered a monodisciplinary record (63/448, 14.0%). The largest group involved colleagues from different disciplines; these were defined as multidisciplinary records (201/448, 44.9%). The highest level of activity was found in the multidisciplinary records (mean 12.2), but even in the solo records there was activity at a mean level of 3.43. The majority of the activities were care activities (mean 9.14; email had a mean of 0.89 and process activities 0.29). In care activities, the action that was performed most frequently was observations (mean 4.09), and other care activities (problem assessment, care action, and care action adaption) were found to be at a mean level of approximately 2. Emailing took place at a mean level of 0.89. Within the category of process activities, “inviting involved colleagues to link,” which is a new action in the care process when using e-communication, took place at a mean level of 1.88. The action that was taken the least was “handing over coordination” (mean 0.29).

In answer to the third question, activity was found to increase with multidisciplinarity within the patient record. Most activities occurred in the multidisciplinary patient records, with a mean number of 12.2 activities per record (Table 1). When professionals worked in monodisciplinary records, the mean number was 6.35, and in solo records it was 3.43.

The conclusion is that Congredi is well used; there is significantly more activity when more disciplines are present in a record, and this is a prerequisite for effective care [21]. The results of this study therefore underscore the feasibility of Congredi to facilitate multidisciplinary communication concerning the care of home-dwelling elderly patients. Congredi might also be feasible in handover situations because different professionals can look at the same record and note their observations and activities. Therefore, every professional is informed of the latest situation. The findings of other studies show that handover situations are a great risk for this population [3,22]. The results of our study show that this risk can be alleviated with a digital communication system, including a patient record. More research is needed to verify whether the quality of care does, in fact, increase.

### Observations Concerning Implementation

Further diffusion of this innovation is promising. A participation rate of 21.4% was achieved, which is quite successful for an innovative intervention. An explanation might be found in Rogers’ theory on diffusion of innovation. He found that in the first phase of diffusion the adoption rate is generally approximately 16%, with innovators and early adopters using it [23]. It is posited that the point at which innovations tend to diffuse in society to the level where they can sustain themselves is when the early and late majorities become active after the innovators and early adopters (16%) [23].

In nearly half of the patient records, multidisciplinary communication about care problems actually took place. This is a high rate. Part of the higher adoption rate in this study could be explained by the regional approach with which the context was managed. It could also be explained by the stepwise implementation based on feedback by the users (choice of communication tool, feasibility study, decision to evaluate the innovation) and support at an administrative level.

When implementing an e-communication tool in primary care, it is interesting to examine not only whether the professionals use the tool but also whether it has potential to support them in their professional work methods. In this study, we found that the care plan was used as it was intended; problems were assessed, actions were defined, observations were noted, and actions were adapted (Plan-Do-Check-Act cycle). Problems were listed in 85% (169/201) of the patients’ records. In most cases, the number of problems during the study period did not increase (in more than half of the cases, only one problem was registered); in a third, there was more than one problem, which could be a signal for higher complexity (Table 2). Care actions were defined in approximately three-fourths of the records; in half of the records more than one action was taken. Observations were found in nearly all the records; sharing them with colleagues is a form of integrating care because professionals can act on the observations of colleagues. Care actions were adapted in over two thirds of the records; in half this took place more than once. This could indicate instability. In conclusion, a relatively active multidisciplinary practice was shown in relation to the duration of the study (10 months).

### New Functionalities in Care Process

Congredi also offers new functionalities for professionals compared with usual care. Understanding how professionals use these functionalities is important for the further implementation of this program.

First, it is now possible for coordinating professionals to actively invite their colleague to link to a mutual patient record. This can be viewed as strengthening the network around the elderly; in this way, the relevant professionals have a direct overview of the situation and can thus take relevant action. This was done by the professionals in more than 90% of the patient records. In combinations with nurses and general practitioners (GN), 2 or more other professionals were invited during the 10 months. Because the relevant colleagues actively shared a care plan, it could be supposed that they perceive this functionality as supportive to their work process.

Second, sharing observations about patients took place on a large scale. Making observations was not new, but the transparency of sharing observations that could influence actions of other professionals was new. The exchange of such relevant information could result in a better-informed professional team, as indicated in the eCCM [12]. Further research could be done to determine whether this has an effect on decreasing the fragmentation of care.

Third, emailing within the patient record was a new function of the e-communication tool, which made it possible to view the care plan and the email communication together. This was expected to be experienced by the professionals as an improvement. Emailing took place in 31.4% of the patient records at a mean level of 0.89. This level was lower than expected, which might be explained by the fact that there are other email channels that are already in use.

All of the functionalities gained by using an e-communication tool are important prerequisites for effective communication among professionals about a patient care plan. This study shows that linking colleagues and sharing observations, which could result in stronger networks and integrated care, appealed to the users the most.

Another finding of this study was that approximately 40% of the professionals, the solo records, did not use Congredi as a multidisciplinary communication tool; they opened patient records but did not invite colleagues to link. Half of this group did, however, perform actions within the patient records. Through some personal communications, an explanation was given that Congredi helped them structure their own work more than the tools they had at their disposal. Because electronic administration tools in home care organizations in the Netherlands are primarily directed at cost administration in contrast to supporting nurses in their nursing work and because by far the largest discipline that worked solo in the care plan was the group of nurses (80%), this might be a motive. Most general practitioners already have an effective electronic administration tool. This could explain why a relatively small group worked alone and why the general practitioners in solo records were less active than the nurses and other professionals. Professionals continually strive for easy access between tools such as Congredi and their own professional administration systems; the feasibility study showed that not having a direct link influenced their motivation to participate actively in multidisciplinary communication. Facilitating work processes logistically should be a focus in further implementation.

### Clarification Needed

During the study, the focus was on whether the professionals would use the tool and were able to use it. This goal was successfully achieved as professionals entered the study and patient records were opened. During the analysis, another question surfaced: Which frequency of actions in an electronic communication tool makes it successful? In other words, what level of activity in the patient records means that the tool is successful within the work process? In this study, the results show quite a variance in the number of actions in multidisciplinary patient records. In some patient records, there was little action, and in others there was much more. It is possible that professionals are just not using the tool. Another reason could be that factors related to the patient’s situation influence the number of communications. Two studies about interprofessional communication in primary care give some indication. An observational study in primary practice stresses the fact that frequent communication through different communication channels is effective [24]. Peeters et al [25] found that there tends to be no interdisciplinary communication if nothing is wrong. The insight that depending on the situation patients rely more or less on the support of professionals could help with implementation. Therefore, if there is little communication in a stable situation, professionals do not need to be disappointed, and when there is deterioration in the patient’s situation, more contact is expected. The findings in the literature also show that patients seem to appreciate the possibility of e-communication with their professional [26].

### Strengths and Limitations

A methodological strength of the study was the large number and diversity of participating professionals and patient records. In addition to a relatively high participation rate, active communication was found among the professionals. As discussed previously, this was mainly due to the management of the context within which the innovation took place.

A limitation was that little comparison with “usual multidisciplinary communication” was found in the literature. It would be interesting to determine how the degree of peer communication within Congredi relates to multidisciplinary communication without Congredi. One study showed some quantification of structural communication on a yearly basis as perceived by the professionals, but because it was not specified per patient, a comparison with this study cannot be made [10].

In this exploratory study of multidisciplinary communication using electronic tools, quantitative data were used; this is an important first step to gain insight into the use of e-communication by professionals. Studying registered data has a limitation. For more insights into barriers and facilitators, qualitative data might be useful.

### Conclusions

In conclusion, Congredi has the potential to improve multidisciplinary communication for home-dwelling elderly patients with 2 or more professional health caregivers. In this study, it was used by a large group of professionals for their patients. Congredi seems to support professional work processes, and it offers new functions that have the potential to improve quality of care. Further research is needed to understand its implementation for different groups of patients.

## Abbreviations

CCM: chronic care model
eCCM: eHealth Enhanced Chronic Care Model
SFMPC: social, functional, mental, physical, and communication
